# Comparison Between Effect of Lidocaine, Morphine and Ketamine Spray on Post-Tonsillectomy Pain in Children

**DOI:** 10.5812/aapm.4092

**Published:** 2012-07-10

**Authors:** Seyed Abbas Hosseini Jahromi, Seyedeh Masoumeh Hosseini Valami, Sevak Hatamian

**Affiliations:** 1 Department of Anesthesiology, Shahid Rajaee Hospital, Qazvin University of Medical Sciences, Qazvin, Iran; 2 Department of Anesthesiology, Hamadan University of Medical Sciences, Hamadan, Iran

**Keywords:** Analgesia, Ketamine, Lidocaine, Morphine, Tonsillectomy

## Abstract

**Background:**

An effective pain therapy to block or modify the physiological responses to stress has become an essential component of modern pediatric anesthesia and surgical practice.

**Objectives:**

The goal of this study was to compare the analgesic effects of the spray forms of; lidocaine, morphine, ketamine, and normal saline on post-tonsillectomy pain scores in children.

**Patients and Methods:**

A total of 120 children, American Society of Anesthesiologist (ASA) class I–II, scheduled for elective tonsillectomy were enrolled in this double-blind, clinical trial study. They were randomly divided into 4 groups, each receiving one of the following drugs at the end of operation; lidocaine spray (2 mg/kg); morphine spray (0.05 mg/kg); ketamine spray (0.5 mg/kg); or normal saline spray (8 puffs). For comparison of postoperative pain; the face, legs, activity, cry, consolability (FLACC) pain scale was evaluated during the first one hour of recovery period at 20 minutes intervals. The data was transferred to SPSS-10 software and analyzed statistically with the Scheffe test and Dunnett’s T3 test. *P* value less than 0.05 was considered significant.

**Results:**

In the early postoperative period (time: 0) and at 20, 40 and 60 minutes of recovery time, FLACC scale scores in the control group were higher than in the three other groups. At 20 minutes, the FLACC scale score in the lidocaine group was lower than in the other groups. At 40 minutes, the statistical differences between FLACC scales of the lidocaine, morphine and ketamine groups were not significant (*P* > 0.05). Finally, at 60 minutes, the FLACC scale scores of the ketamine and morphine groups were lower than in the other groups.

**Conclusions:**

The results of this study showed that lidocaine spray had the best pain controlling effect at 20 minutes in the recovery room, but after 40 minutes, ketamine and morphine sprays were more effective than the lidocaine spray.

## 1. Background

Pain is an unpleasant sensory and emotional experiences associated with actual or potential tissue damage (1, 2).

Undergoing treatment may result in the occurrence of post-operative pain and this triggers biochemical and physiological stress responses. It also causes impairments in; pulmonary, cardiovascular, neuroendocrinal, gastrointestinal, immunological, and metabolic functions (2, 3). Children are special in this regard, since in this group it is a very complex phenomenon. It is very difficult to differentiate restlessness or crying due to pain, hunger or fear.

An effective pain therapy to block or modify the physiological responses to stress has become an essential component of modern pediatric anesthesia and surgical practice (2).

The Society for Pediatric Anesthesia, clearly defined the alleviation of pain as a “basic human right”, irrespective of age and medical condition (4).

Realistic aims for pediatric post-operative pain management are to recognize pain in children, to minimize moderate and severe pain safely in all children, to prevent pain where it is predictable, to bring pain rapidly under control and to continue pain control after discharge from hospital (5–10).

Tonsillectomy is a common pediatric surgical procedure associated with significant postoperative pain that is a challenge to treat (11–15). Inadequate pain management after tonsillectomy may result in; poor oral intake, dehydration, sleep disturbances, behavioral changes, and emesis (13, 15) and many other side effects as previously mentioned. It was suggested that early and frequent oral intake in the postoperative period significantly reduces postoperative pain (16).

Analgesic spray is a novel administration for postoperative pain control. It is delivered by a pump or pressurized container. Such sprays are topical, meaning that they are applied to the surfaces of the body, most commonly the skin, but sometimes to the mucous membranes, such as the throat. A common reason for using an analgesic spray or other topical painkiller, rather than a painkiller taken by mouth, is that topical treatments work directly on the affected area. Topical administration of analgesic drugs such as morphine produces a localized analgesic effect in inflamed skin or mucosal tissue. Transnasal butorphanol spray (a synthetic opioid agonist-antagonist drug) produces rapid pain alleviation in patients undergoing oropharyngeal surgery (17).

Farhex spray (chlorhexidine gluconate 0.12% and benzydamine HCl 0.15%) is a topical agent that exhibits antiseptic, anti-inflammatory, and analgesic effects, it is used for postoperative pain treatment in tonsillectomy. In this study researchers concluded that pain scores in the study group were lower than in the control group at different times (18).

## 2. Objectives

Therefore, we conducted the present study, in which we compared the efficacies of four different medications in spray form including; lidocaine, morphine, ketamine, and normal saline for decreasing post-tonsillectomy pain scores in children.

## 3. Patients and Methods

After approval by the Medical Ethics Committee and obtaining written informed consent from the parents of the participants, 120 children, American Society of Anesthesiologists (ASA) I–II (age, 3–12 years) scheduled to undergo elective tonsillectomy in a pediatric hospital in Qazvin, Iran, were enrolled in this double-blind, controlled clinical trial study. They were randomly assigned (using colored cards) to; lidocaine, morphine, ketamine, and normal saline groups.

This study was powered on the basis of previous results showing a 50% incidence of post-tonsillectomy pain in the control group. A sample size of 30 patients in each group was calculated to detect a decrease in the incidence of post-tonsillectomy pain down to 15% with α = 0.05 and β = 0.2.

Patients with a positive history of asthma, drug allergy to lidocaine or morphine, and consumers of anticonvulsive, analgesic or anti-inflammatory drugs were excluded from this study.

For premedication, 0.02 mg/kg midazolam, 1 μg/kg fentanyl and 0.02 mg/kg atropine were administered intravenously to all patients. They also received 5 mg/kg sodium thiopental and 0.5 mg/kg atracurium intravenously for induction of anesthesia and isoflurane (1%) with O2 and N2O (50% – 50%) during the operation for maintenance of the anesthesia. At the end of the operation, one anesthesiologist applied spray forms of lidocaine 10% (2 mg/kg), morphine 0.1% (0.05 mg/kg), ketamine 1% (0.5 mg/kg) or normal saline (8 puffs) in the tonsillar fossa.

After deep extubation and transferring the patient to the recovery room, a blinded researcher carried out assessment of postoperative pain by the; face, legs, activity, cry, consolability (FLACC) pain scaling system for one hour at twenty-minute intervals.

The FLACC pain scaling system is a behavioral pain assessment scale, which is used to assess pain in children or individuals who are unable to communicate their pain. The scale has 5 criteria and each is assigned a score of; 0, 1 or 2. It is scored between a range of 0–10 with 0; representing no pain, relaxed and comfortable, 1–3; mild discomfort, 4–6; moderate pain, 7–10; severe discomfort or pain or both (19).

In our study, in patients with a FLACC score of 4 or over, we used intravenous meperidine (0.5 mg/kg) for the treatment of postoperative pain.

All data was transferred to SPSS-10 software and analyzed statistically with Scheffe test and Dunnett’s T3 test. *P* value less than 0.05 was considered significant.

The clinical trial registration number is: IRCT201104236256N1

## 4. Results

Demographic data (age, sex and weight) were not significantly different between the four groups (*Table 1*).

**Table 1:** Patients’ Demographic Characteristics

Variable	Groups				*P* value
	Ketamine, n = 30	Morphine, n = 30	Lidocaine, n = 30	Placebo, n = 30	
Age, y, mean ± SD	6.5 ± 2.3	6.5 ± 2.1	6.6 ± 2.1	6.6 ± 2.3	> 0.05
Weight, Kg, mean ± SD	21.1 ± 6.4	20.5 ± 5.8	21.9 ± 6	21.3 ± 6.1	> 0.05
Sex, No.					> 0.05
Male	14	16	17	13	
Female	16	14	13	17	

Immediately after the patients transfer to the recovery room, there was no significant statistical difference between the FLACC scores of patients in the morphine or the control group, but the difference was significant between the; ketamine-control, lidocaine-control and ketamine-lidocaine groups (*Table 2*).

**Table 2:** FLACC Scale Differences Between Four Groups at the Onset of the Recovery Period (Time: 0)

Group	Mean Difference	Std. Error	*P* value
Control *vs* Ketamine	1.048	0.226	0.000
Control *vs* Morphine	0.124	0.248	0.997
Control *vs* Lidocaine	2.866	0.177	0.000
Ketamine *vs* Lidocaine	1.818	0.201	0.000

**Figure 1:**
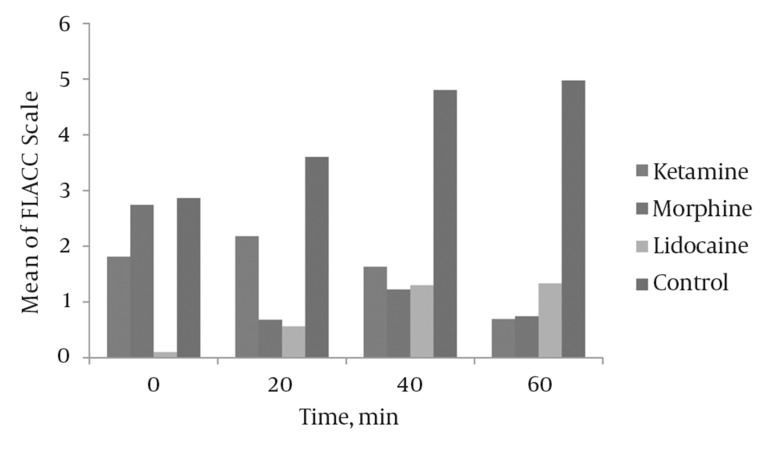
Mean of FLACC Scale at Different Times in the Study

In the early postoperative period (time: 0) and at 20, 40 and 60 minutes of recovery time, the FLACC scores in the control group were higher than in the three other groups (*Figure 1*).

We demonstrate the differences in the FLACC scale scores between the four study groups at 20, 40 and 60 minutes of recovery period in *Table 3*.

**Table 3:** FLACC Scale Differences Between Four Groups at 20, 40, and 60 Minutes of Recovery Time

Group	20 Minutes			40 Minutes			60 Minutes		
	Mean Difference	Std. Error	*P* value	Mean Difference	Std. Error	*P* value	Mean Difference	Std. Error	*P* value
Control *vs* Ketamine	1.418	0.232	0.000	3.436	0.213	0.000	4.303	0.177	0.000
Control *vs* Morphine	1.438	0.235	0.000	3.574	0.217	0.000	4.258	0.180	0.000
Control *vs* Lidocaine	3.033	0.237	0.000	3.500	0.218	0.000	3.666	0.181	0.000
Ketamine *vs* Morphine	*0.020*	0.230	1.000	0.137	0.211	0.935	0.044	0.176	0.996
Ketamine *vs* Lidocaine	1.615	0.232	0.000	0.063	0.213	0.993	0.636	0.177	0.007
Morphine *vs* Lidocaine	1.594	0.235	0.000	0.074	0.217	0.990	0.591	0.180	0.016

**Figure 2:**
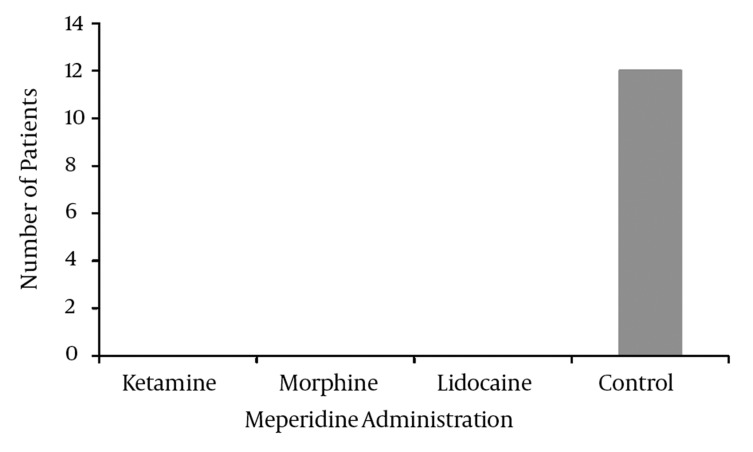
Number of Patients Who Received Meperidine in Different Groups

Patients in the lidocaine, morphine and ketamine groups did not have a FLACC scale score of 4 or more, and therefore they did not receive meperidine, but intravenous meperidine was administered to twelve patients in the control group (*Figure 2*).

## 5. Discussion

In our study, ketamine, morphine and lidocaine sprays were more effective than a placebo for controlling postoperative pain, but these three medications had a different action onset.

At the beginning of the recovery period, lidocaine produced the best analgesia, ketamine led to lesser analgesia than lidocaine, but morphine did not have any analgesic effect at this time.

At 20 minutes, lidocaine still produced the best analgesia, but ketamine and morphine produced similar analgesia effects. At 40 minutes, the three medications had the same effect in producing analgesia, but at 60 minutes, morphine and ketamine produced the most significant analgesia.

The main limitation of our study was its short duration. In order to increase the accuracy of the study results, we instructed only one researcher to evaluate the FLACC scale scores. We thought that a longer study duration may create a bias due to the assessment of FLACC scores by more than one researcher.

Tonsillectomy produces large areas of exposed muscle in the oropharynx, resulting in considerable pain from muscle spasm and irritation of the nerve endings. Also excessive dissection and use of cautery-hemostasis may produce an even larger amount of inflammation and post-operative pain (20).

The most important factors which determine postoperative outcomes are known to be; incisional pain, nausea and vomiting, preoperative anxiety and discomfort from intravenous injection. It has been determined that more than 60% of children who have had an operation exhibit negative behavioral changes in the two weeks after this experience (21–24). Thus, it is very important to provide effective postoperative analgesia in children.

The development of alternative methods of drug administration has improved the ability of physicians to manage specific problems. Practitioners recognize the rapid onset, relative reliability, and the general lack of patient discomfort when drugs are administered by the transmucosal and transdermal routes. They have administered sedatives, narcotics, and a variety of other medications by; transdermal, sublingual, nasal, rectal, and even tracheal-mucosal routes in a variety of practice settings (25).

Drug absorption through a mucosal surface is generally efficient because the mucosal surfaces are usually rich in blood supply, providing the means for rapid drug transport to the systemic circulation and avoiding, in most cases, degradation by first-pass hepatic metabolism (25, 26).

The oral transmucosal route has been used for many years to provide rapid blood nitrate levels for the treatment of angina pectoris. The fentanyl buccal tablet was developed to take advantage of oral transmucosal absorption for the painless administration of an opioid in a formulation acceptable to children (25).

In one study by Akbay *et al.* that investigated the analgesic efficacy of topical tramadol in the control of postoperative pain in children after tonsillectomy, they concluded that topical 5% tramadol with its local anesthetic effect seems to be an easy, safe, and comfortable approach for pain management in children undergoing tonsillectomy (27).

In another study by Atef and Fawaz concerning the effect of intravenous paracetamol, they concluded that it provides rapid and effective analgesia in tonsillectomy (28).

In several studies about the reduction of post-tonsillectomy pain by local infiltration of bupivacaine, the authors concluded that marcaine can produce effective analgesia (29, 30).

In conclusion, the present study found that, lidocaine spray is suitable for producing early analgesia; however, morphine spray and ketamine spray lead to later analgesia. Therefore, we suggest that future researchers study the analgesic effects of the combination of lidocaine spray with one of the morphine or ketamine sprays, in order to find better methods for producing post-tonsillectomy analgesia in children.
